# Lyophilized annelid mega-hemoglobin retains its’ quaternary structure and oxygen equilibrium properties after room temperature storage for over 6 months

**DOI:** 10.1371/journal.pone.0263996

**Published:** 2022-02-17

**Authors:** Chintan Savla, Andre F. Palmer

**Affiliations:** William G. Lowrie Department of Chemical and Biomolecular Engineering, The Ohio State University, Columbus, Ohio, United States of America; Indian Institute of Technology Kharagpur, INDIA

## Abstract

The long-term storage stability and portability of hemoglobin (Hb)-based oxygen carriers are important design criteria in the development of these therapeutics. Lyophilization or storing proteins in a freeze-dried form is known to increase storage lifetime and reduce overall weight. In this study, we lyophilized the extracellular mega-hemoglobin of the annelid *Lumbricus terrestris* and tested the storage stability at different temperatures and oxygenation conditions. Storage in refrigerated conditions for over 6 months in the presence of N_2_ reduced oxidation by 50% while storage at room temperature in the presence of N_2_ reduced oxidation by 60%, all while maintaining the structural stability of the mega-hemoglobin. We also demonstrated a reliable strategy to freeze dry Hbs in the presence of a minimally non-reducing disaccharide sugar that could be easily re-solubilized in deionized water. Overall, this study made significant advances towards long term storage stability of oxygen therapeutics for direct applications in transfusion medicine.

## Introduction

The storage stability of blood and blood products is a key area of concern for researchers and medical professionals. Hemoglobin (Hb)-based oxygen carriers (HBOCs) are a class of red blood cell (RBC) substitute that typically rely on chemically modifying or encapsulating human, bovine, and annelid Hbs to improve their biocompatibility for *in vivo* use. Strategies to chemically modify or encapsulate Hb include surface conjugated (PEGylated) Hb [1–4], intermolecularly cross-linked and polymerized Hb [5], vesicle and liposome encapsulated Hb (LEHs) [6], and coprecipitated or desolvated Hb nanoparticles [7, 8]. To reduce the formation of methemoglobin (metHb) in these HBOC formulations, storage conditions for HBOCs include freezing at -80°C, storage at 4°C, and room temperature storage under deoxygenated conditions. While lyophilization was used in the past to store liposome encapsulated Hbs [9–11], freeze drying has seen a resurgence for storage of next generation HBOCs [12–14]. Storage of these HBOCs in freeze dried form not only increases their storage life, but also reduces overall HBOC weight, which increases their portability. Given the urgent need for blood products in combat medicine, a low-weight, portable, and easily reconstituteable HBOC is desirable. In this study, we assessed if lyophilization was a viable storage strategy for the mega-hemoglobin (erythrocruorin, Ec) derived from the annelid, *Lumbricus terrestris* (Lt). The LtEc mega-Hb is comprised of iron-centered porphyrin groups in each of its 4 subunits within the Hb tetramer; 12 (tetramers) of which are held together using linker proteins to form the hexagonal bilayer structure of LtEc. The LtEc mega-structure has 144 heme groups and is ~30 nm in diameter, which prevents extravasation of the molecule from the vascular space into the tissue space. Apart from its large size that prevents tissue extravasation, hypertension, and oxidative tissue stress, LtEc is stable under physiological conditions, exhibits moderate affinity for oxygen similar to human RBCs, exhibits a lower auto-oxidation rate than human Hb (hHb), and can be easily surface PEGylated to increase its’ circulatory life [15]. Studies to test the *in vivo* biocompatibility of LtEc have confirmed its’ reduced vasoactivity, and thus support the use of LtEc in transfusion medicine applications [16, 17]. These properties make it an excellent candidate for use as an artificial RBC substitute. While we do know a lot about the storage stability of LtEc at 4°C, storage post lyophilization has not been studied before. In this study, we lyophilized LtEc and hHb, and monitored metHb formation, structural stability, and equilibrium oxygen binding properties after 6 months of storage under various temperature conditions and in the presence and absence of a deoxygenated environment.

## Experimental section

### Materials

Expired human RBC units were generously donated by Transfusion Services, Wexner Medical Center, The Ohio State University, Columbus, OH. Batches of 1000 Canadian night crawlers were purchased from Wholesale Bait Company (Hamilton, OH). Trehalose (C_12_H_22_O_11_), sodium phosphate dibasic (Na_2_HPO_4_), sodium phosphate monobasic (NaH_2_PO_4_), sodium chloride (NaCl), potassium chloride (KCl), sodium hydroxide (NaOH), and tris-HCl, were purchased from Sigma-Aldrich (St. Louis, MO). Hollow fiber (HF) tangential flow filtration (TFF) modules of MW cut-offs (MWCO) 0.5 μm PES, 0.22 μm PES, 500 kDa PS, and 50 kDa PS were purchased from Repligen Co., Waltham, MA. All other chemicals and supplies were purchased from Fisher Scientific, Pittsburgh, PA.

### hHb and LtEc purification

hHb was purified using TFF from expired human RBCs as described previously in the literature [18]. In short, expired RBC units were lysed using 3.75 mM phosphate buffer (PB) pH 7.4 and purified on a multistage TFF system using 0.2 μm and 50 kDa HF filter modules. LtEc was purified using TFF from *Lumbricus terrestris* worms as described previously in the literature [19]. In short, 1000 Canadian Nightcrawlers were blended in Tris-HCl buffer, and the homogenate was sequentially centrifuged at 3700 ×g and 18000 ×g to separate worm debris and particulates, respectively. The cloudy supernatant was clarified using multistage TFF HF modules of MWCO 0.5 and 0.22 μm. The purified product was diafiltered using phosphate buffer saline (PBS, 0.1 M pH 7.4) over a 500 kDa HF membrane to remove smaller proteins and concentrated to ∼100 mg/ mL for subsequent storage at −80°C.

### Lyophilization

Hb solutions were concentrated to ~50 mg/mL and trehalose was added as a lyoprotectant (concentrations 0.1, 0.25, 0.5 M) before freezing them at -80°C for subsequent lyophilization. Frozen samples were dried under reduced pressure using a Labconco FreeZone 2.5L benchtop freeze dryer for 24 hours. Lyophilized samples were stored at 4°C, 25°C, and -80°C in the presence and absence of N_2_ for 6 months.

### Total and metHb measurements

Total Hb and metHb concentrations were determined using the cyanmethemoglobin method [20]. Spectrophotometric absorbance measurements were obtained using a HP 8452A diode array spectrophotometer (Olis, Bogart, GA).

### Quaternary structure (SEC-HPLC)

hHb and LtEc samples were separated on an analytical Acclaim SEC-1000 (4.6 × 300 mm) column (Thermo Fisher Scientific, Waltham, MA) as previously described in the literature [19]. The mobile phase consisted of 50 mM sodium phosphate buffer (PB) at pH 7.4. Chromeleon 7 software was used to control HPLC parameters such as flow rate (0.35 mL/min), UV-visible absorbance detection (280 nm [to detect total protein] and 413 nm [to detect heme]). All samples were filtered through 0.2 μm syringe filters (Titan3, Thermo Fisher Scientific, Waltham, MA) before size exclusion (SEC) HPLC analysis.

### Oxygen (O_2_) equilibrium curves

O_2_ equilibrium curves (OECs) were measured using a Hemox Analyzer (TCS Scientific Corp., New Hope, PA). Protein samples were diluted to ~60 μM (heme basis) in 5 mL Hemox buffer (pH 7.4) with 20 μL of Additive A, 20 μL of Additive B, and 20 μL of antifoaming solution (TCS Scientific). The temperature was maintained at 37.0 ± 0.1°C. The Hill equation was used to fit the OEC data, and the P_50_ (partial pressure of O_2_ at which the Hb is half saturated with O_2_) and n (cooperativity coefficient) parameters were regressed from the curve fits [21].

### Statistical analysis

In this study, an ANOVA test was performed to determine if multiple groups were statistically different followed by a Tukey post-hoc test to determine the groups that differed from one another. A p value of < 0.05 was considered statistically significant.

## Results and discussion

**Fig 1:**
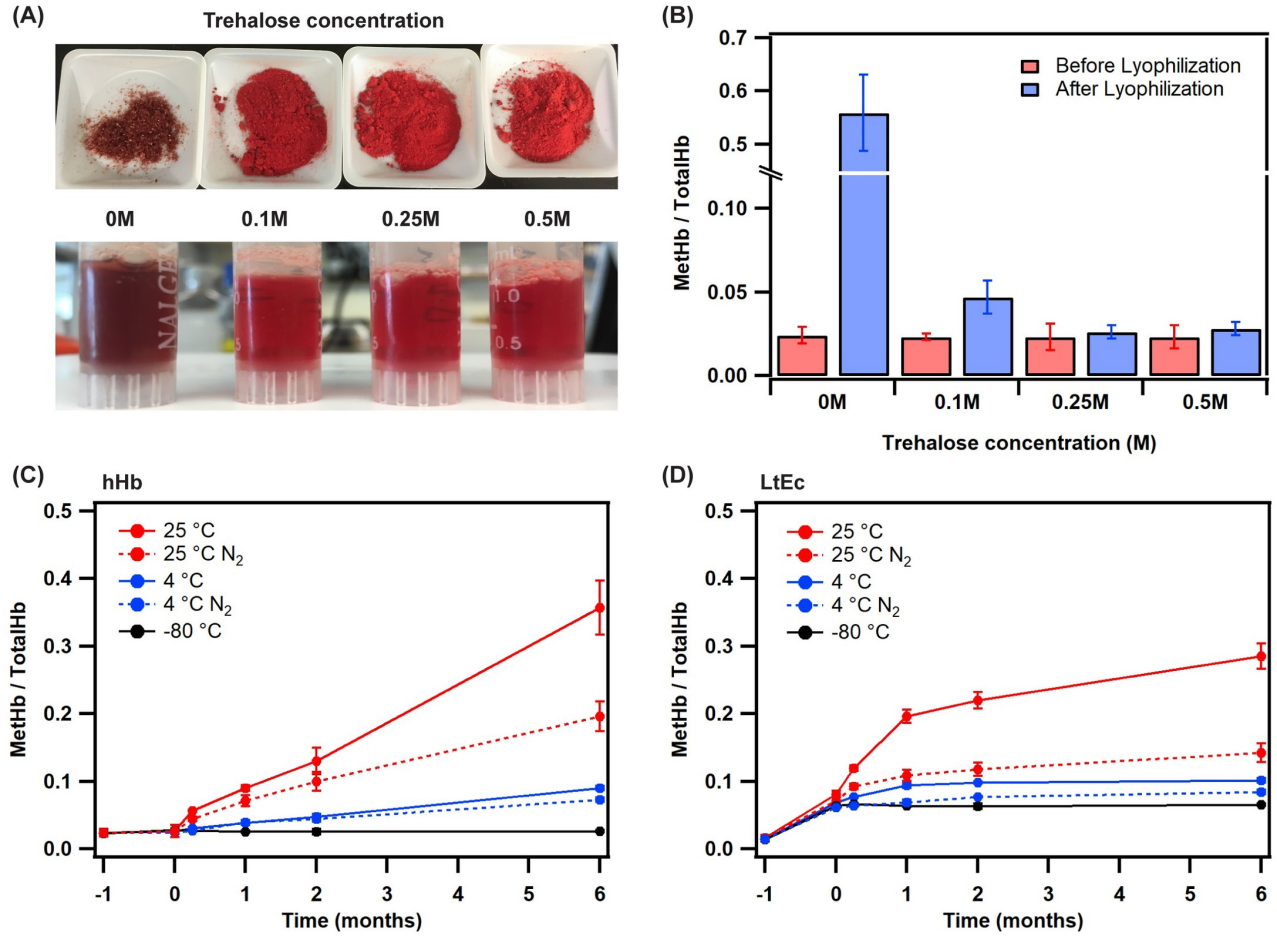
(A) Images of hHb after lyophilization in powdered and rehydrated form as a function of trehalose concentration. (B) MetHb formation in hHb samples before and after lyophilization as a function of trehalose concentration. (C-D) MetHb formation for hHb and LtEc samples respectively over 6 months. Conditions tested were storage at 25°C, 4°C and -80°C in the presence and absence of a N_2_ environment (i.e., oxygenated/deoxygenated conditions). Error bars indicate the standard deviation from the mean.

Human Hb (hHb) was used as a control to test the effect of lyoprotectant concentration on the protein before and after the lyophilization process. Cryo-protectants such as glycerol, polyethylene glycol (PEG), disaccharides, etc. have been used in the literature to protect proteins during the harsh freeze-drying process [22, 23]. Disaccharides have been known to stabilize biological molecules, preserve enzymatic activity, and prevent thermal denaturation of proteins [11]. For this study, we chose to use trehalose (a non-reducing disaccharide) as studies have shown it to be an effective cryo-protectant for Hb-based species [11, 12]. In the absence of trehalose, the lyophilized Hb powder turned dark brown in color indicative of metHb formation (Fig 1A). As the concentration of trehalose increased from 0.1 M to 0.5 M, the Hb powder turned bright red and had a grainy texture. All Hb powders were easily soluble in deionized water after mixing for <30 secs (Fig 1A). The lyophilized Hb with 0 M of trehalose had >55±6% conversion to non-functional metHb. For 0.1 M trehalose, the metHb level was ~5±2%, which further reduced to 3±1% with 0.5 M trehalose (Fig 1B). Based on this data, 0.1 M trehalose was chosen as the concentration of cryo-protectant for all future studies, as it provided ample protection against metHb formation during the freeze-drying process and limited the amount of disaccharide sugar in the final product. Further, previous studies with freeze-dried plasma have shown that ~10% w/v sugar concentration was sufficient to prevent protein aggregation and reduce the accumulation of reactive oxygen species [24, 25]. Hb and LtEc samples were stored under different temperature and gas conditions post lyophilization for 6 months and the formation of metHb was monitored over time—ambient temperature (25°C), cold storage (4°C), and freezer storage (-80°C) in the presence and absence of a N_2_ atmosphere. Storing HBOCs under deoxygenated conditions is known to reduce the oxidation of Hb to non-functional metHb, and hence it was important to test the storage stability in the presence of N_2_. Directly after post lyophilization, hHb samples did not undergo substantial metHb formation, whereas LtEc samples exhibited an increase in metHb level from 2±0% to 6±1% (Fig 1C and 1D). This is consistent with our previous results which show that LtEc undergoes ~2–3% increase in metHb level per freeze-thaw cycle [19]. Furthermore, during storage at 25°C over 6 months, the metHb level for hHb increased from 2±0% to 35±4% in the absence of N_2_ and 18±2% in the presence of N_2_. It is important to note that the Hb was not deoxygenated prior to exposure to N_2_, which explains the high metHb level even under N_2_ storage. In comparison, the metHb level for LtEc rose from 6±1% to 29±2% in the absence of N_2_ and increased to 12±1% in the presence of N_2_. At 4°C, the metHb level for hHb increased from 2±0% to 8±1% in the absence of N_2_ and increased to 7±1% in the presence of N_2_, whereas for LtEc it increased from 6±1% to 10±2% in the absence of N_2_ and increased to 9±1% in the presence of N_2_. At -80°C, no significant change was observed in metHb for either species. These results suggest that the rate of metHb formation for LtEc was much slower (~1.5 ×) than that of hHb indicating that LtEc could be stored for longer durations at ambient temperature and 4°C than hHb (and subsequently other hHb derivatives). This was consistent with the auto-oxidation results previously reported by our group. LtEc exhibited an auto-oxidation rate of 0.004 h^-1^, whereas hHb had a rate of 0.013 h^-1^ at 37°C which is ~3× faster [19]. These experiments concluded that storing both Hb species at 4°C in the presence of N_2_ was ideal. However, storing lyophilized LtEc at room temperature under deoxygenated conditions for up to 6 months is also a viable option for use in emergency use such as ambulances, battlefield, etc.

**Fig 2:**
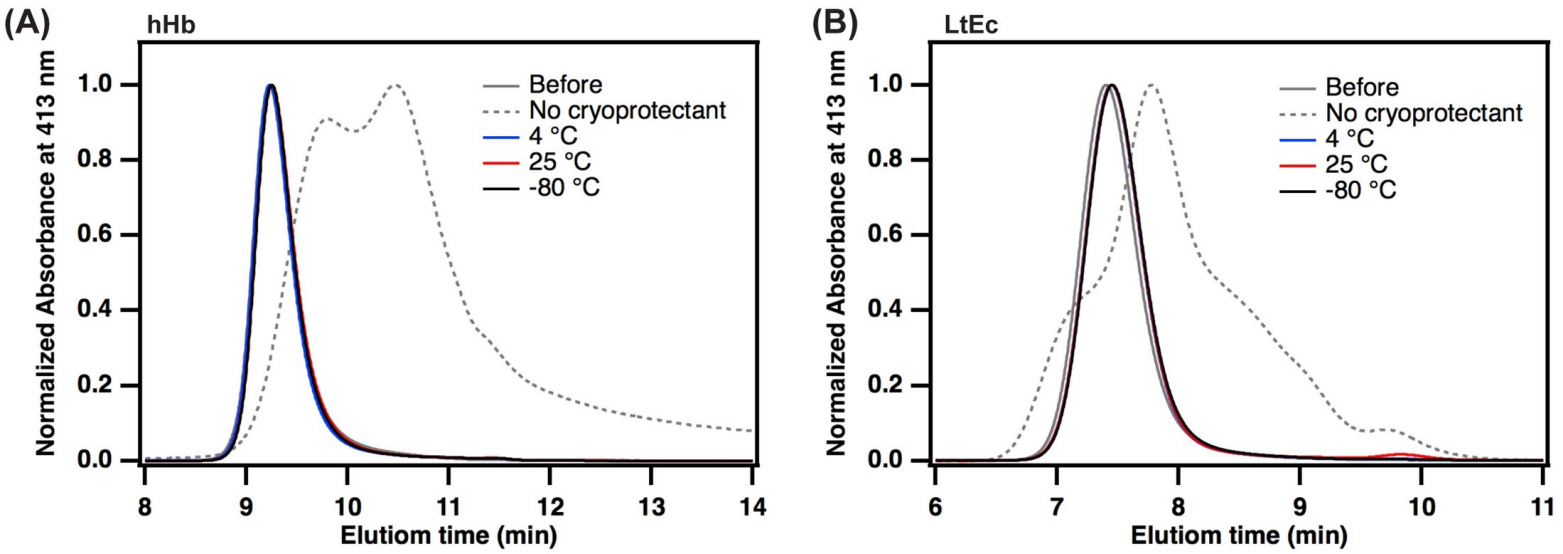
SEC-HPLC analysis of (A) hHb and (B) LtEc samples post lyophilization stored under different conditions—no cryoprotectant, with cryoprotectant at temperatures of 25°C, 4°C, and -80°C. All SEC-HPLC curves are after 6 months of storage under the respective storage conditions.

To further monitor the quaternary structure of the Hb species before and after lyophilization, SEC-HPLC analysis was performed (Fig 2A and 2B). Separations were performed over an Acclaim SEC-1000 column with continuous UV-visible absorbance measurements at 280 nm (protein peak) and 413 nm (heme peak). The SEC-HPLC separates components based on their size resulting in larger molecules eluting first from the column, with smaller molecules eluting at a later time. The maximal elution time peak for native hHb was 9.2 mins (MW~64 kDa), whereas for LtEc it was 7.6 mins (MW~3.6 MDa) owing to its large megahemoglobin structure consisting of 12 protomeric units connected via linker proteins. In the absence of a lyoprotectant, we observed that Hb dissociated into two species that eluted at 9.7 mins (αβ dimers) and 10.6 (α and β monomers) mins respectively with peak broadening indicative of protein denaturation. Similarly, LtEc dissociated into protomers, linkers, and monomers along with unknown peaks indicative of denaturation of the mega-protein structure. This result, along with the presence of brown crystals and high metHb formation in the absence of lyoprotectant confirmed the importance of adding trehalose before the freeze-drying process. Furthermore, no change in the elution time or peak width was observed for any Hb species at all temperature conditions with lyoprotectant. This was an important result as it verified the integrity of the quaternary structure of LtEc after 6 months of storage with lyoprotectant at all temperatures.

**Fig 3:**
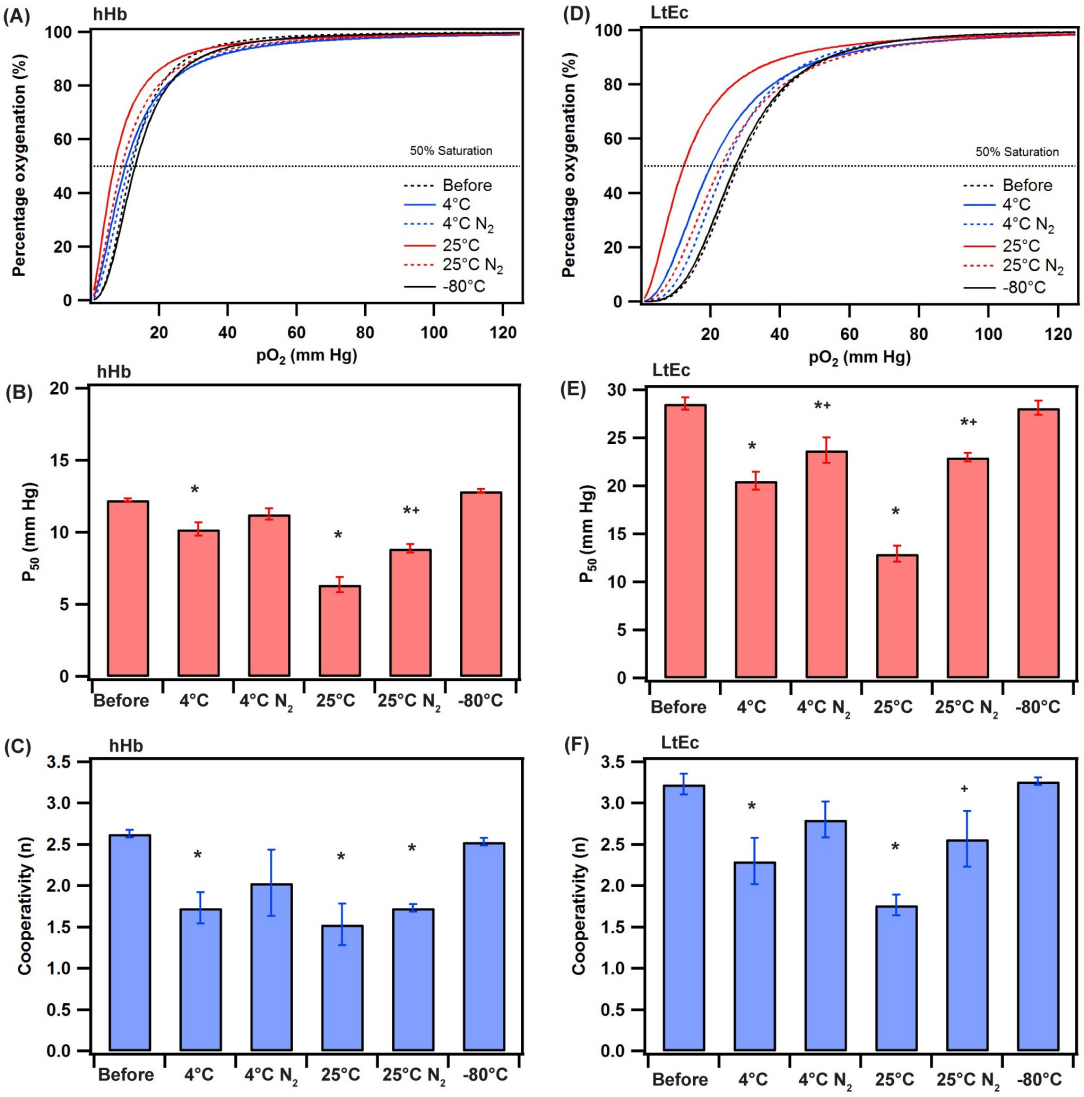
(A, D) Oxygen equilibrium curves of hHb and LtEc pre and post lyophilization stored for 6 months under different conditions. (B, E) Partial oxygen pressure at 50% O_2_ saturation of hHb or LtEc (P_50_). (C, F) Cooperativity coefficient (n). * p<0.05 compared to before lyophilization. **+** p<0.05 compared to the same temperature condition in the absence of N_2_.

To evaluate the equilibrium oxygen binding properties of the Hbs post lyophilization, oxygen equilibrium curves were measured for all Hbs using a HEMOX analyzer (Fig 3). The HEMOX analyzer is used to control the partial pressure of O_2_ (pO_2_) in solution and measures the O_2_ saturation of the Hb as a function of pO_2_. The partial pressure of O_2_ at which the Hb is 50% saturated with O_2_ (i.e., the O_2_ affinity, P_50_) and cooperativity coefficient (n) were determined by fitting the OEC data to the Hill equation. For hHb, a one-way ANOVA test determined that temperature had a significant impact on the P_50_ (p<0.0001) and n (p<0.001). Before freeze drying, the P_50_ and n for hHb was 12±0.09 mm Hg and 2.6±0.04, whereas for LtEc it was 28±0.6 mm Hg and 3.2±0.1, respectively. After storing hHb at 4°C for 6 months, the P_50_ reduced to 9±0.3 mm Hg in the absence of N_2_ (significantly different than before) and 11±0.4 mm Hg in the presence of N_2_ (not significantly different). Similarly, for LtEc, a one way ANOVA test determined that temperature had a significant impact on the P_50_ (p<0.0001) and n (p<0.01). Also, a similar trend was observed for LtEc with the P_50_ reducing to 20±0.9 mm Hg in the absence of N_2_ (significantly different than before) and 24±1.3 mm Hg in the presence of N_2_ (significant different than the no N_2_ groups). Similarly, after storing for 6 months at 25°C, the P_50_ for hHb and LtEc saw a significant reduction in the absence of N_2_ with a slight increase while in the presence of N_2_ but still significantly different from before and no N_2_ groups. In the presence of N_2_, the reduction in P_50_ observed was far lower at 4°C and 25°C for both Hb species further confirming the need to store Hb under deoxygenated conditions for a longer storage life and preservation of Hb properties. The cooperativity followed similar trends to P_50_ with the Hbs stored at 4°C preserved more cooperativity than Hbs stored at 25°C while the Hbs stored in the presence of N_2_ had higher cooperativity than those stored in the absence of N_2_.

## Conclusion

In conclusion, we demonstrated a reliable strategy to add minimal amounts of non-reducing disaccharide (trehalose) to hHb and LtEc samples and freeze drying them to obtain a bright red powder. This powder was easily re-solubilized in deionized water in under 30 secs and had a UV-visible spectrum without any scattering. Based on the metHb level formed after lyophilization, 0.1 M trehalose was determined to be sufficient to maintain the structural stability (SEC-HPLC) and O_2_ binding properties (metHb assay) of Hb. After storing lyophilized Hb powders under different temperature and gas conditions, we found that the Hbs retained their quaternary structure, oxygen equilibrium properties, and >80% oxygen carrying capacity when stored for up to 6 months at 4°C in the presence of N_2_. Since it may not be possible to always store materials in a refrigerated environment, storing Hbs at 25°C in the presence of N_2_ also retained >80% of the oxygen carrying capacity after 6 months, while still maintaining the oxygen equilibrium properties of the materials. Previous studies by our group have already concluded that LtEc is a reliable oxygen carrier *in vivo* that maintains microcirculatory parameters, oxygen transport to tissues, and eliminates problems associated with Hb extravasation due to its large molecular size [19]. Taken together, lyophilized LtEc powder stored at 4°C or 25°C in the presence of N_2_ can maintain its biochemical and biophysical properties for up to 6 months and hence is a solid candidate for use as an RBC substitute in remote locations and in the absence of an immediate supply of RBC units.

## Supporting information

S1 Data (XLSX)
